# Supplementary material for: Identification of photoperiod-induced specific miRNAs in the adrenal glands of Sunite sheep (Ovis aries)
Source: Front Vet Sci. 2022 Jul 22;9:888207. doi: 10.3389/fvets.2022.888207 (PMC9354845; doi:10.3389/fvets.2022.888207)
Supplement: Supplementary file 1 [file Data_Sheet_1.ZIP › Supplementary file/miRDeep2 file.zip!/miRDeep2 file/Know_Structure/SP42A3/oar-mir-409.pdf]

miRBase precursor : oar-mir-409
Total read count : 9921
oar-miR-409-5p read count : 1876
oar-miR-409-3p read count : 8044
remaining reads : 1

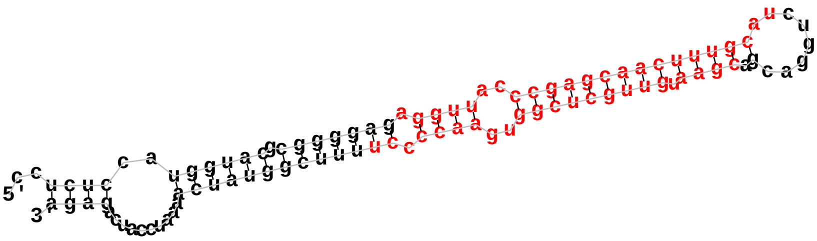

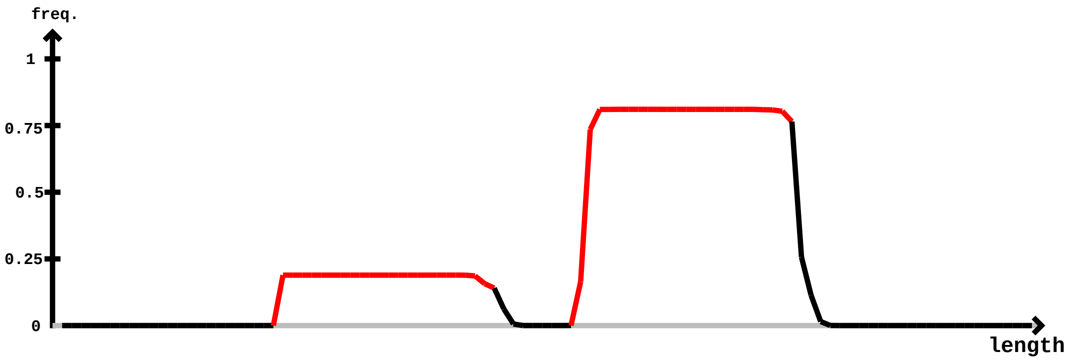


```
                                    oar-miR-409-5p
                                                               oar-miR-409-3p
5'- ccucuccaugguacgcggggagagguuacccgagcaacuuugcaucuggacgacgaauguugcucggugaaccccuuuucgguaucaaaauccaucagaga   -3'    exp
    .((((...((((((.(((((((.((((..((((((((((((((........).)))).)))))))))..)))).)))))))))))))).........)))).    reads    mm    sample
    ......................agguuacccgagcaacuuu............................................................    2        0     seq
    ......................agguuacccgagcaacuuug...........................................................    29       0     seq
    ......................agguuacccgagcaacuuugc..........................................................    286      0     seq
    ......................agguuacccgagcaacuuugca.........................................................    165      0     seq
    ......................agguuacccgagcaacuuugcau........................................................    777      0     seq
    ......................agguuacccgagcaacuuugcauc.......................................................    557      0     seq
    ......................agguuacccgagcaacuuugcaucu......................................................    59       0     seq
    .......................gguuacccgagcaacuuugca.........................................................    1        0     seq
    ......................................uuugcaucuggacgacgaauguu........................................    1        0     seq
    ....................................................acgaauguugcucggugaac.............................    2        0     seq
    .....................................................cgaauguugcucggugaac.............................    8        0     seq
    .....................................................cgaauguugcucggugaacc............................    6        0     seq
    .....................................................cgaauguugcucggugaaccc...........................    12       0     seq
    .....................................................cgaauguugcucggugaacccc..........................    133      0     seq
    .....................................................cgaauguugcucggugaaccccu.........................    999      0     seq
    .....................................................cgaauguugcucggugaaccccuu........................    339      0     seq
    .....................................................cgaauguugcucggugaaccccuuu.......................    114      0     seq
    .....................................................cgaauguugcucggugaaccccuuuu......................    19       0     seq
    ......................................................gaauguugcucggugaac.............................    3        0     seq
    ......................................................gaauguugcucggugaacc............................    4        0     seq
    ......................................................gaauguugcucggugaaccc...........................    32       0     seq
    ......................................................gaauguugcucggugaacccc..........................    231      0     seq
    ......................................................gaauguugcucggugaaccccu.........................    3531     0     seq
    ......................................................gaauguugcucggugaaccccuu........................    970      0     seq
    ......................................................gaauguugcucggugaaccccuuu.......................    775      0     seq
    ......................................................gaauguugcucggugaaccccuuuu......................    116      0     seq
    ......................................................gaauguugcucggugaaccccuuuuc.....................    2        0     seq
    .......................................................aauguugcucggugaaccc...........................    5        0     seq
    .......................................................aauguugcucggugaacccc..........................    18       0     seq
    .......................................................aauguugcucggugaaccccu.........................    513      0     seq
    .......................................................aauguugcucggugaaccccuu........................    111      0     seq
    .......................................................aauguugcucggugaaccccuuu.......................    85       0     seq
    .......................................................aauguugcucggugaaccccuuuu......................    10       0     seq
    .......................................................aauguugcucggugaaccccuuuuc.....................    1        0     seq
```

```
                      oar-miR-409-5p
                                                       oar-miR-409-3p
ccucuccaugguacgcggggagagguuacccgagcaacuuugcaucuggacgacgaauguugcucggugaaccccuuuucgguaucaaaauccaucagaga

........................................................auguugcucggugaaccccuu........................    1    0    seq
.........................................................uguugcucggugaaccccuuu.......................    3    0    seq
.........................................................uguugcucggugaaccccuuuu......................    1    0    seq
```
